# Pathophysiological analysis of serially measured plasma biomarkers, remote monitoring and clinical events, in symptomatic patients with moderate to severe chronic heart failure

**DOI:** 10.1016/j.ijcha.2026.101970

**Published:** 2026-07-11

**Authors:** Y. Allach, M. Barry - Loncq de Jong, P.R.D. Clephas, S. Abou Kamar, H.P. Brunner-La Rocca, M.L. Handoko, V.P. van Halm, W.E.M. Kok, F.W. Asselbergs, R.R.J. van Kimmenade, S.L.M.A. Beeres, M. Rienstra, M.K. Szymanski, R.A. de Boer, I. Kardys, J.J. Brugts

**Affiliations:** a Department of Cardiology, Cardiovascular institute, Erasmus University Medical Center, Rotterdam, Netherlands; b Department of Cardiology, Cardiovascular Research Institute Maastricht (CARIM), Maastricht University, Maastricht, Netherlands; c Department of Cardiology, Utrecht University Medical Centre, Utrecht, Netherlands; d Department of Cardiology, Amsterdam Cardiovascular Sciences, Amsterdam University Medical Centre, University of Amsterdam, Amsterdam, Netherlands; e Department of Cardiology, Radboud University Medical Centre, Nijmegen, Netherlands; f Department of Cardiology, Leiden University Medical Centre, Leiden, Netherlands; g Department of Cardiology, University of Groningen, University Medical Centre Groningen, Groningen, Netherlands

**Keywords:** Temporal, Heart failure, Congestion, Serial measurements, Biomarker

## Abstract

**Aims:**

To explore temporal patterns of serially measured cardiovascular-related biomarkers in patients with chronic heart failure (CHF), with the objective of identifying biomarker pathophysiological trajectories associated with clinical outcomes. These explorative analyses can generate hypotheses regarding underlying pathophysiological processes and the potential role of multi-biomarker approaches in chronic HF.

**Methods:**

The BioMEMS-study involved 334 patients with moderate to severe chronic HF in NYHA class III who received either standard of care or remote hemodynamic monitoring. Serial blood samples were collected at baseline, 3, 6, and 12 months, and biomarker levels were assessed using the Olink Cardiovascular-III panel. Joint modelling analyses were performed, integrating longitudinal biomarker trajectories and risk of the composite endpoint of all-cause mortality or HF hospitalization.

**Results:**

In multivariable-adjusted models, 15 biomarkers were consistently and significantly associated with the composite endpoint after adjustment for confounders and multiple testing. MMP-2, ST2, IGFBP-1, IGFBP-7, and NT-proBNP exhibited the strongest associations with the composite endpoint, with hazard ratios (95% CI) of 2.72 (1.87–4.03), 2.71 (2.00–3.80), 2.70 (1.81–4.27), 2.48 (1.81–3.45), and 2.28 (1.72–3.03), respectively. These findings were robust across sensitivity analyses correcting for clinical confounders and treatment groups. Temporal trajectories revealed higher biomarker levels in patients who experienced incident events, with biomarkers showing corresponding changes in levels preceding events.

**Conclusion:**

Serial biomarker measurements could provide additional insights in the pathophysiology of worsening HF. Specific biomarkers reflecting myocardial stress, cardiac remodelling and fibrosis show changes in levels over time as worsening HF approaches, which highlights possible involvement of these pathophysiological pathways.

## Highlights

• Serial biomarker measurements improve insights in pathophysiological mechanisms of worsening HF in CHF patients.
• 15 biomarkers, including MMP-2, ST2, and NT-proBNP were related to adverse events.
• Temporal biomarker trajectories and multimarker panels could potentially improve individualized HF management.

## Graphical abstract

**Figure:**
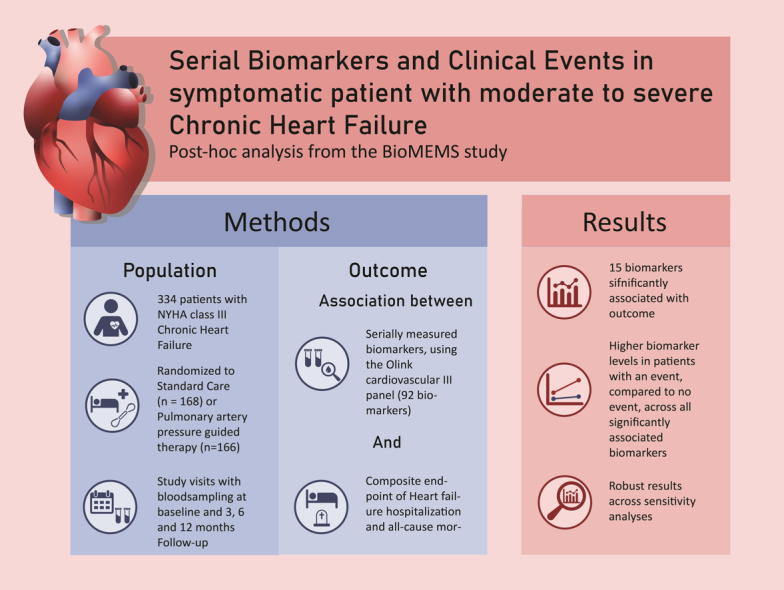

## 1 Introduction

Increased levels of plasma biomarkers involved in pathophysiological pathways, including neurohormonal activation, myocardial and extracellular matrix (ECM) remodelling, and inflammation, have demonstrated diagnostic and prognostic value in heart failure (HF) progression [1], [2], [3]. The clinical value appears stronger when using serial measurements of circulating biomarkers over time, rather than using single-point assessments [4], [5]. These serial measurements allow for the identification of trends in biomarkers over time and for taking into account the full, individual temporal patterns for pathophysiological assessments. Circulating biomarkers, reflecting underlying processes in HF, are expected to change with disease progression and before adverse events occur [1], [6], [7]. Insights in these changes could improve our understanding of underlying pathophysiological processes in HF progression. Since existing studies generally cover short periods of time, data on the temporal dynamics of biological markers during the progression to worsening HF are limited [8], [9]. Moreover, most studies measure only one or a few biomarkers. Serial measurements of multiple biomarkers may provide more prognostic information and better understanding of the pathophysiology and disease trajectory in HF.

We aimed to investigate the relationship between repeatedly measured cardiac biomarkers and clinical outcomes in patients with chronic HF. We conducted serial assessments of 92 cardiovascular disease-related human protein biomarkers (OLINK CV-III panel). Our primary objective was to elucidate how these temporal biomarker patterns and their fluctuations correlate with clinical outcomes. In addition, we aimed to generate hypotheses regarding underlying pathophysiological mechanisms and the potential role of multi-biomarker approaches and risk-profiling in chronic HF. Importantly, this study does not provide a clinical prediction model.

## 2 Methods

### 2.1 Study design and population

The BioMEMS study is an explorative sub-study of the MONITOR-HF trial (clinical registration number: NTR7673 (NL7430)) and investigates the association between serially measured biomarkers and clinical events. We previously published the rationale and study design, including a complete list of eligibility-criteria [10], [11]. This investigator-initiated study was a prospective, multicentre, randomised clinical trial involving patients with symptomatic moderate to severe chronic HF in NYHA functional class III with a previous HF admission in the past 12 months, treated with contemporary guideline recommended medical therapy in the Netherlands. Study participants (*n* = 348) were randomised in a 1:1 ratio to receive either remote hemodynamic monitoring by pulmonary artery pressure guided therapy on top of standard of care or standard of care alone (Supplementary table 1). In the BioMEMS study, two blood samples (EDTA plasma and serum) were obtained from each participant at baseline and at study visits at 3, 6, and 12-months. Details on blood sample collection and storage are described elsewhere [11]. For this post-hoc analysis, 334 patients were available who received their allocated treatment and had at least one blood sample drawn (166 and 168 respectively in each study arm). The study protocol received approval from the central Medical Ethics Review Committee (METC 2018–2563) as well as from the institutional review boards of all participating sites. Written informed consent was obtained from all participants, and the study adhered to the principles outlined in the Declaration of Helsinki.

### 2.2 Biomarker analyses

The Cardiovascular Target 96 Panel III (Olink Proteomics AB, Uppsala, Sweden) was utilized to measure biomarkers in EDTA plasma samples. All analyses were conducted at the Olink Core Facility of the University Medical Centre Utrecht, the Netherlands. The Olink panel assay utilizes proximity assay technology to analyse 92 high-abundance proteins [12].

**Fig. 1:**
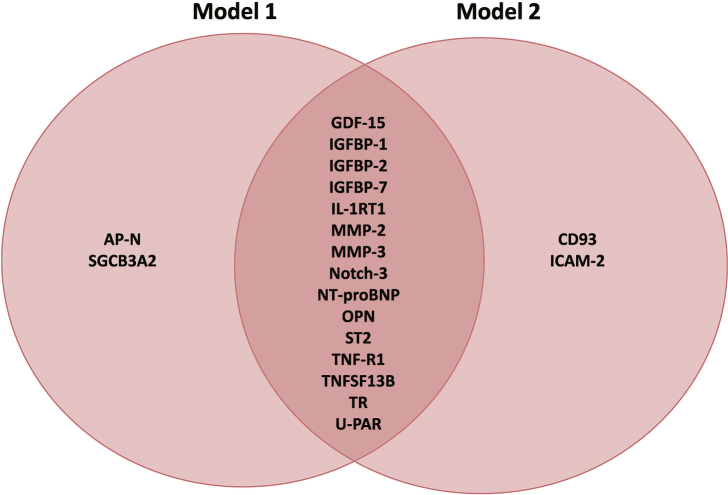
Overview of the serially measured blood biomarkers significantly associated with the composite endpoint, using joint models with two different correction models. The first correction model (Model 1) accounted for age, sex, study arm, duration of HF, hypertension, estimated glomerular filtration rate (eGFR), and the use of guideline-directed medical therapy (GDMT) at baseline. The second model (Model 2) included these variables as well as additional adjustments for atrial fibrillation (AF), left ventricular ejection fraction (LVEF), and body mass index (BMI). For the longitudinal component of the joint model (the linear mixed-effect model), adjustments were made for age, sex, duration of HF, and eGFR. All analyses were corrected for multiple testing (Benjamini Hochberg).

Biomarker concentrations are expressed by Olink Proteomics as normalized protein expression (NPX) values on a log2 scale, offering a semi-quantitative comparison of protein levels, with higher NPX values indicating relatively elevated protein levels in the samples [12]. A 1-unit increase on this log2 scale corresponds to a doubling of the protein level. Values were normalized to internal controls or reference samples within the study population, ensuring that the reported protein levels are relative to a standardized reference expressions level. Ten samples did not meet quality assessment criteria and were therefore excluded from subsequent analysis. Furthermore, two of the 92 biomarkers (SPON-1 and TLT-2) were excluded from further analysis because over 50% of their measurements fell below the Lower Limit of Detection (LOD). Patients with at least one valid biomarker measurement were included in the analysis (334 patients). A mean of 3.1 samples (SD 1.0) meeting all quality criteria were available per patient (148 patients with 4 biomarker measurements, 102 patients with 3 measurements, 49 patients with 2 measurements, and 35 patients with one measurement), yielding a total of 1031 samples for the analysis of 90 biomarkers (Fig. 1).

### 2.3 Clinical outcome

The clinical outcome of this biomarker substudy and the presented analyses is an composite endpoint, which included HF hospitalizations, urgent visits requiring intravenous diuretics, or all-cause mortality, whichever occurred first [13]. We used a time frame of one year of follow-up, in which biomarkers samples were drawn at regular outpatient follow-up visits at baseline, 3, 6, and 12 months. The clinical events were assessed in this year of follow-up up to six weeks after the 12 months visit.

### 2.4 Statistical analysis

Baseline data were summarized, using the median and 25th to 75th percentile, or count with percentages, as appropriate. In order to enable direct comparisons between biomarkers, a *Z*-score of the log2-transformed biomarker values, as expressed in NPX was used. Associations between serial biomarker levels and the composite endpoint, were assessed using joint modelling analyses. These analyses consisted of linear mixed-effects models to estimate individual temporal trajectories of each biomarker over time, which are estimated jointly with a relative risk model, to assess their associations with the risk of reaching the composite endpoint. In the joint models, the composite endpoint was treated as the dependent variable, while the serially measured biomarker levels served as independent variables. Each biomarker was analysed separately in the joint model, resulting in a total of 90 single-biomarker joint models. In the Cox proportional hazard models, used as part of the joint modelling framework, two correction models were applied to adjust for potential confounding factors that may differ between patients with and without an event. These models were based on existing literature as well as factors that were significantly different between outcome groups at baseline. Correction model 1 accounted for age, sex, study arm, duration of HF, hypertension, estimated glomerular filtration rate (eGFR), and the use of guideline-directed medical therapy (GDMT), specifically the four drug classes: Renin-angiotensin-aldosterone system inhibitors, beta-blockers, mineralocorticoid receptor antagonists, and sodium-glucose cotransporter-2 inhibitors at baseline. Correction model 2 included these variables as well as additional adjustments for atrial fibrillation (AF), left ventricular ejection fraction (LVEF), and body mass index (BMI). For the linear mixed-effect models, time was used as the independent variable, biomarker level was the dependent, and random intercepts and slopes were included to account for individual variations in biomarker trajectories over time, capturing heterogeneity in temporal trends among patients. Splines were used if necessary. Adjustments were made for age, sex, duration of HF, and eGFR. Both correction models were applied to the entire study cohort, involving both treatment arms. Hazard ratios for biomarkers identified as significantly associated with the composite endpoint were displayed in a forest plot. To illustrate the temporal dynamics of biomarkers significantly associated with the endpoint, effect plots were generated. These plots depict the longitudinal trajectories of biomarker levels, stratified by patients who reached the endpoint and those who did not. Additionally, we evaluated whether evolvement of serial biomarker levels over time was influenced by treatment group. This analysis compared associations of biomarkers with outcome, between the intervention and control groups by repeating the joint modelling analysis described above in both groups, using correction model 1 (due to lower number of events and group sizes). For six biomarkers, including CD93, CDH5, GRN, IL-17RA, IL-1RT1, and NOTCH-3, the use of splines in this model was not possible due to complexity of the models. To assess whether the associations between biomarker trajectories and outcome differed between treatment groups, we calculated *p*-values for the interaction terms between biomarker trajectories and treatment allocation in the full cohort.

Finally, a sensitivity analysis was conducted using a more elaborate linear mixed effects model, in order to check for robustness of the results. In this sensitivity analysis, correction model 2 was expanded with a linear mixed-effects model adjusting for age, sex, study arm, duration of HF, hypertension, eGFR, use of GDMT, AF, LVEF and BMI, in the full patient cohort. For one biomarker, IL-1RT1, the use of splines was not possible due to complexity of the model.

Statistical significance was defined as a two-sided *p*-value <0.05 for analysis of baseline characteristics. Remaining analyses were adjusted for multiple testing using the Benjamini-Hochberg method, with a false discovery rate (FDR) <0.05. All data analyses were performed using R (version 4.3.1.).

## 3 Results

The baseline characteristics of study participants, stratified by the occurrence of an event, are presented in Table 1**.**

**Table 1:** Baseline characteristics in patients with or without clinical event.

	No event (*n* = 203)	Event (*n* = 131)	
			P-value
Age (median (IQR))	69.5 [59.1, 75.0]	69.2 [63.2, 75.5]	0.43
Male sex (n, %)	154 (75.9)	97 (74.0)	0.81
Previous myocardial infarction (n, (%))	83 (40.9)	59 (45.0)	0.53
Diabetes (n, (%))	76 (37.4)	51 (38.9)	0.87
Cerebrovascular accident or transient ischaemic attack (n, (%))	37 (18.2)	28 (21.4)	0.57
Atrial fibrillation (n, (%))	102 (50.2)	70 (53.4)	0.65
Hypertension (n, (%))	109 (53.7)	86 (66.2)	**0.03**
eGFR, mL/min (median (IQR))	52.0 [39.0, 67.0]	43.0 [34.0, 54.0]	**<0.001**
Body mass index, kg/m2 (median (IQR))	27.2 [24.4, 32.0]	26.8 [24.0, 30.2]	0.26
Left ventricular ejection fraction <40% (n, (%))	150 (73.9)	91 (70.0)	0.52
Heart failure etiology = non-ischemic (n, %)	100 (49.3)	67 (51.1)	0.82
Years since heart failure diagnosis (median [IQR])	3.1 [0.8, 8.5]	5.0 [1.3, 8.7]	0.09
Months since last heart failure hospitalization (median [IQR])	4.2 [1.8, 8.1]	3.0 [1.3, 5.3]	**0.01**
Remote hemodynamic monitoring treatment (n, %)	107 (52.7)	59 (45.0)	0.21
Beta blockers (n (% yes))	177 (87.2)	102 (77.9)	**0.04**
Renin-angiotensin-aldosterone system inhibitor (n (% yes))	182 (89.7)	94 (71.8)	**<0.001**
Mineralocorticoid receptor antagonist (n (% yes))	172 (84.7)	102 (77.9)	0.15
SGLT2 inhibitor (n (% yes))	19 (9.4)	12 (9.2)	1.00
Diuretics (n (% yes))	193 (95.1)	128 (97.7)	0.35

Data are presented as n (%) or median [25th–75th percentile]. eGFR; estimated glomerular filtration rate. Renin-angiotensin-aldosterone system inhibitor includes Angiotensin-converting enzyme inhibitor, Angiotensin-receptor blocker and Angiotensin-receptor neprilysin inhibitor. Diuretics include Loop diuretics and Thiazide diuretics.

### 3.1 Temporal patterns of biomarkers in relation to the composite clinical endpoint

**Fig. 2:**
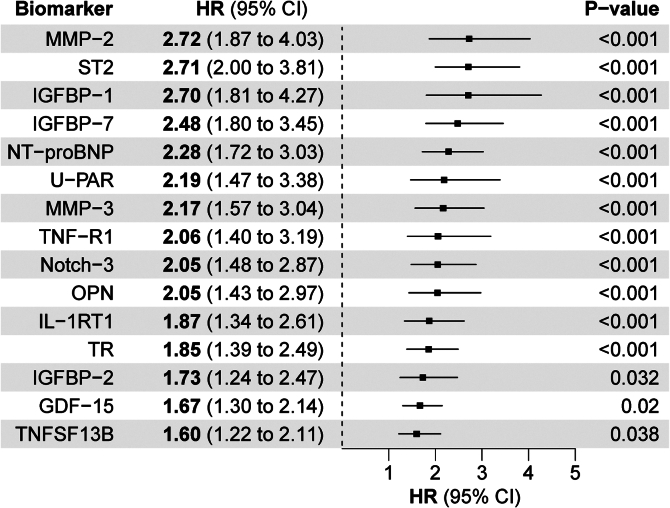
Forest plots of the associations between serially measured blood biomarkers significantly associated with the composite endpoint, using a joint model. The correction model accounted for age, sex, study arm, duration of HF, hypertension, estimated glomerular filtration rate (eGFR), the use of guideline-directed medical therapy (GDMT) at baseline, atrial fibrillation (AF), left ventricular ejection fraction (LVEF), and body mass index (BMI). For the longitudinal component of the joint model (the linear mixed-effect model), adjustments were made for age, sex, duration of HF, and eGFR. All analyses were corrected for multiple testing (Benjamini Hochberg).

**Fig. 3:**
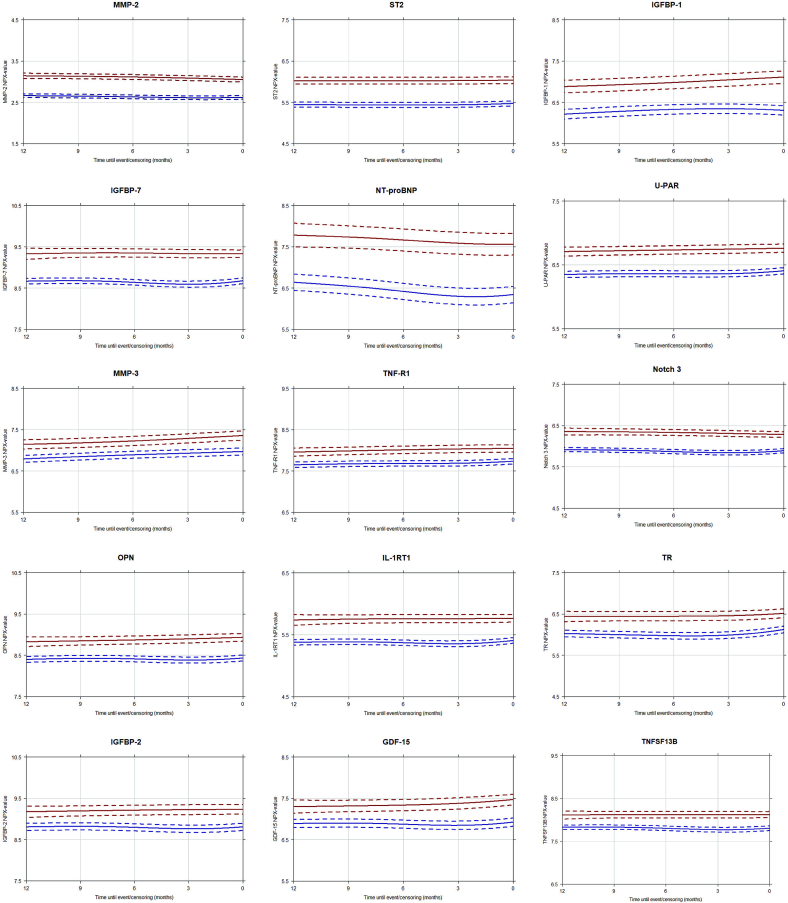
The temporal dynamics of biomarkers significantly associated with the endpoint. The average trajectories of circulating biomarkers during the year preceding a primary endpoint event, comparing patients who experienced the event with those who remained event-free. The solid red line represents the average biomarker trajectory in patients who reached the endpoint, while the solid blue line indicates the trajectory for those who remained event-free. Dashed lines depict the 95% confidence intervals for each group.

The relationship between serially measured blood biomarkers and the composite study endpoint, was assessed using joint modelling, incorporating two correction models to adjust for potential confounders in the Cox proportional hazards framework. The first model, adjusted for age, sex, study arm, duration of HF, hypertension, eGFR, and the use of GDMT at baseline, identified 17 biomarkers significantly associated with the composite endpoint (Fig. 1, supplementary table 2). The second model, which additionally accounted for AF, LVEF and BMI, yielded 17 significantly associated biomarkers (supplementary table 3**)**. Fifteen of these biomarkers occurred in both models and four biomarkers were not shared between the models, namely AP-N, CD93, ICAM-2 and SGCB3A2 (Fig. 1). The 15 overlapping biomarkers are presented in Fig. 2, including the hazard ratios as identified in the second, more comprehensive correction model. Matrix metalloproteinase – 2 (MMP-2), Interleukin 1 receptor like 1 (ST2), Insulin-like Growth Factor Binding Protein 1 (IGFBP-1), Insulin-like Growth Factor Binding Protein 7 (IGFBP-7), and N-terminal Prohormone Brain Natriuretic Peptide (NT-proBNP) exhibited the strongest associations with the composite endpoint, with hazard ratios (95% CI) of 2.72 (1.87–4.03), 2.71 (2.00–3.80), 2.70 (1.81–4.27), 2.48 (1.81–3.45), and 2.28 (1.72–3.03), respectively. Temporal trajectories of biomarker levels of the 15 overlapping biomarkers are visualized in effect plots (Fig. 3). These plots demonstrate that the biomarker values were significantly higher in individuals who experienced an event. Furthermore, for several biomarkers, such as IGFBP-1 and GDF-15, an increase in level was observed during the time preceding the event.

### 3.2 Subgroup analysis

When comparing the intervention and control groups and testing for interactions between biomarkers and treatment, we did not find significant interactions (all *p*-values for interaction were non-significant). Among patients who received the remote hemodynamic monitoring on top of standard care, five biomarkers showed a significant association with the composite endpoint, namely GDF-15, ICAM-2, IGFBP-7, OPN and ST2 (supplementary table 4). These biomarkers were also significantly associated with the composite endpoint in the main analysis (total study population). Among the patients who received standard care, eight biomarkers met this threshold. These were IGFBP-1, IGFBP-7, MMP-2, MMP-3, NT-proBNP, ST2, TNFRSF10C, and TR. All these biomarkers, except TNFRSF10C, overlap with the significantly associated biomarkers in the main analysis considering the total study population.

### 3.3 Sensitivity analysis with expanded linear-mixed effects modelling

The sensitivity analysis in the full cohort, using correction model 2 with the expanded linear mixed-effects model adjusting for age, sex, study arm, duration of HF, hypertension, eGFR, use of GDMT, AF, LVEF and BMI yielded 20 significantly associated biomarkers (supplementary table 5).

## 4 Discussion

In this study, we examined serially measured cardiac biomarkers in relation to clinical outcome, and demonstrated an association between 15 biomarkers and clinical events in a population of symptomatic moderate to severe chronic HF patients. These findings provide insight into possible underlying pathophysiological pathways.

Among these biomarkers, MMP-2, ST2, IGFBP-1, IGFBP-7 and NT-proBNP demonstrated the strongest associations with clinical HF-related events in the total patient population (Fig. 2). No interaction was observed according to treatment group. The effect plots demonstrate that the NPX values of biomarkers significantly associated with the composite endpoint are elevated in patients experiencing an incident event, compared to those who do not. This difference is present from the beginning of follow-up, suggesting that patients with higher risk show higher plasma levels of these biomarkers over a prolonged period. Notably, several biomarkers exhibit a further increase shortly before the incident event. Likewise, NT-proBNP shows a decrease in the mean NPX value in the group of patients who do not experience an event [13]. This study shows overlap with our previous findings, which identified an association between serially measured mean PA pressure (mPAP) readings and biomarkers in the same population [12]. Nine of the biomarkers that were significantly associated with the composite endpoint in this study were previously found to be also associated with mPAP. These were MMP-2, ST2, IGFBP-7, NT-proBNP, U-PAR, Notch 3, TR, GDF-15, and TNFSF13B (supplementary fig. 1). Additionally, the prior study demonstrated that a subset of four biomarkers, exhibited a correlation between longitudinal changes in biomarker levels and changes in mPAP, of which most notably NT-proBNP and MMP-2 overlap with the current study. The overlap shows that a shared set of biomarkers can be associated with both mPAP, and worsening HF in the current study. Identifying such biomarkers could enable better understanding of pathophysiological processes underlying clinical deterioration, as most of the identified biomarkers are related to pathophysiological pathways involved in myocardial stress, cardiac remodelling, extracellular matrix remodelling and fibrosis. The understanding of these processes could potentially lead to biomarker-guided therapy using serial biomarker assessments as a guidance-tool in early risk stratification and eventually therapeutic decision-making in patients with HF. While biomarkers such as MMP-2, ST2, IGFBP-7 and NT-proBNP were significantly associated with HF-related events and pulmonary hemodynamics, it is important to highlight that these biomarkers are not entirely disease-specific, which highlights the need for further research into their disease specificity. To the best of our knowledge, this study is the first to incorporate serial biomarker measurements in a unique cohort of patients with NYHA-class III HF, in whom information on pulmonary artery pressure monitoring was available. In a previous study, serial measurements of biomarkers were analysed in patients with chronic HF [14], that identified e.g. NT-proBNP, ST2, vWF, FABP4, IGFBP1, PAI-1, TR, CHIT1, and PON3, and demonstrated discriminative ability for development of adverse clinical events. NT-proBNP is the cornerstone biomarker in HF management, reflecting myocardial stress and elevated intracardiac pressures [15]. It is an important predictor of HF, demonstrated to be associated with mortality, hospitalization and disease progression [4], [16], [17]. Moreover, higher plasma concentration of NT-proBNP is strongly associated with higher mPAP [[12], [18]]. The possibility of biomarker-guided therapy based on NT-proBNP has also been investigated [19], [20], [21]. However, results from these studies do not show unequivocal results, so there is no role yet in current guidelines [22]. Nevertheless, NT-proBNP remains valuable for clinical decision-making and risk stratification in HF. ST2 is another well-known biomarker in HF prognostication, associated with myocardial fibrosis and remodelling [8], [23]. Elevated levels of ST2 are correlated with worse outcomes in HF, and prognostic value appears less affected by e.g. eGFR and age than other biomarkers, although e.g. infection greatly confounds ST2 interpretation [24]. IGFBP-1 shares biological pathways with IGFBP-2 and IGFBP-7, both of which were significantly associated with the clinical endpoint in this study. These biomarkers play an important role in the pathophysiology of HF progression through mechanisms such as cell growth, apoptosis, and metabolic regulation. IGFBP-1 and IGFBP-2 modulate insulin-like growth factor activity, which influences myocardial remodelling and metabolic dysregulation, both hallmarks of HF progression [[25], [26], [27]]. IGFBP-7 is linked to fibrotic processes and endothelial dysfunction, contributing to elevated filling pressures and cardiac dysfunction in HF [[24], [28], [29]]. MMP-2 plays a role in extracellular matrix degradation, a process essential to HF progression [30], [31]. Higher MMP-2 levels are associated with the development of myocardial fibrosis, leading to stiffness in the heart resulting in increased filling pressures. Moreover, increased MMP-2 activity is associated with worse clinical outcomes in HF patients [32], [33]. GDF-15, is a marker of inflammation and oxidative stress, further emphasizing the relevance of these pathophysiological processes in HF populations [1], [23], [34], [35].

Multiple studies suggest that a multimarker approach may be more appropriate for estimating prognosis than predictive models using single markers [36], [37]. Different biomarkers may reflect distinct biochemical pathways, each affecting the pathophysiology and progression of HF in its own way [17]. A multimarker model can better account for the complexity of the disease and intra- and interpatient variability. In this study, a set of biomarkers is presented that is significantly associated with clinical outcomes; some of them overlap with biomarkers related to mPAP. Since an increase in mPAP may serve as an early indicator of hemodynamic congestion, an important marker of HF-progression, this overlap may indicate a shared pathophysiological mechanism. Although biomarker-guided therapy based on NT-proBNP alone has failed to demonstrate clear improvement in clinical endpoints in large trials, a multimarker approach potentially offers greater efficacy and precision in risk stratification and therapeutic decision-making [19], [21]. Moreover, previous studies have demonstrated that serial biomarker measurements provide more robust prognostic information compared to single time-point assessments. The management of CHF benefits from repeated biomarker measurements that reflect disease progression over time. Importantly, biomarker levels obtained closer to clinical events are generally more strongly associated with outcomes. However, in routine clinical practice, the exact timing of such events is inherently unpredictable. In this context, serial biomarker measurements may provide added value by capturing gradual pathophysiological changes preceding overt clinical deterioration. Future research must determine whether such approaches are clinically relevant, cost-effective and applicable within the framework of personalized and possibly remote (remote) managed care strategies in HF.

### 4.1 Strengths and limitations

One strength of this study is the integration of serial biomarker measurements in a well-phenotyped cohort of moderate to severe HF patients, with information on PAP, allowing for early assessment of congestion and its association with clinical endpoints, enhancing the clinical relevance of our findings. This offers the possibility of identifying biomarkers involved in the pathophysiological processes leading to HF progression. Additionally, the structured design, with serial biomarker measurements over a 12-month follow-up period provides a more informative pathophysiological assessment than single or dual measurements. Finally, the strength of our results is reinforced by multiple sensitivity analyses addressing potential biases by ensuring that the observed biomarker trajectories were not influenced by the differential monitoring strategies employed in the study arms. These confirmed the consistency of biomarker associations across different analytical approaches. This strengthens confidence in the validity of our findings and minimizes potential biases related to differential monitoring strategies between study arms. Several limitations of this study need to be acknowledged. First, this study involves an explorative post hoc analysis in a relatively small cohort, possibly leading to power-issues, especially within treatment subgroups with fewer samples and events. Given the limited number of events relative to the number of biomarkers analysed, there is an existing risk of overfitting that needs to be acknowledged. Consequently, some biomarkers may have achieved statistical significance by chance. To mitigate this, we applied correction for multiple testing using the Benjamini–Hochberg method to control the false discovery rate. However, residual risk of false-positive findings remains, especially in subgroup analyses, and the identified associations should therefore be considered hypothesis-generating and require validation in larger cohorts. The significantly associated biomarkers in the sub-analysis were, however, consistent with those identified in the main analysis. Second, given the multifactorial nature of HF, the complex interaction between biomarkers, hemodynamics, and underlying pathophysiological processes including fibrosis, metabolic dysregulation, and inflammation cannot be fully disentangled or explained based on the results of this study. Moreover, despite multiple correction methods and sensitivity analyses, residual confounding cannot be fully excluded. We adjusted for baseline medication use and assigned treatment group, because both were considered to potentially influence biomarker trajectories and clinical outcomes. However, all therapy changes during follow-up could not be accounted for in our models due to complexity. In a sensitivity analysis, we assessed the interaction between treatment groups and biomarker patterns, but did not find any significant interaction. To this point, it should be added that the study population was restricted to symptomatic HF patients in NYHA class III with a recent history of HF hospitalization and the study was performed in the Netherlands. As a result, the generalizability of our findings may be limited. Extrapolation of these results to other HF populations, including patients with milder symptoms, different HF phenotypes, or other clinical settings, should therefore be made with caution. Third, these findings should be considered explorative and hypothesis generating, rather than confirmatory of specific pathophysiological pathways. This study reflects statistical correlations rather than a direct etiological or causal link between circulating biomarker levels and clinical events. Nevertheless, this approach may indicate relevant pathophysiological pathways warranting further investigation. Given the multifactorial nature of HF, these findings should be interpreted within the context of complex interacting processes. It should be noted that this study represents structured serial biomarker measurements in the outpatient setting and there may be an underestimation of prognostic value in an acute or clinical setting, where biomarkers can fluctuate widely over a short period of time. As biomarker measurements were performed at predefined time points in an outpatient setting, dynamic changes during acute HF events could not be captured, as no blood samples were collected during hospitalizations. Consequently, transient biomarker fluctuations in response to acute decompensation may have been missed. Lastly, the use of the Olink CV-III panel presents some limitations. Biomarker levels are expressed as (semiquantitative) NPX values, rather than absolute concentrations, limiting comparisons with clinical cut-off values and makes it more challenging to draw conclusions regarding prognostic assessment.

### 4.2 Perspective

The association of these biomarkers with HF-related outcomes in this study, that remained consistent over different correction methods, may underscore their future potential value for pathophysiological insights. Each biomarker provides possible insight into distinct pathophysiological pathways, offering a more comprehensive understanding of HF progression. Moreover, our findings align with previous studies investigating biomarker associations with clinical events, as well as our own research on the relationship between biomarkers and mPAP. This overlap suggests that a panel of biomarkers may serve as a tool for insights in disease progression. Future research should focus on refining and validating a multi-biomarker panel capable of generating patient-tailored pathophysiological and prognostic profiles that could help in risk stratification and resources allocation. By integrating multiple, serially measured biomarkers, this approach could enable more precise risk prediction. In general, such an individualized biomarker-driven strategy holds promise for optimizing HF management and improving patient outcomes. However, it is yet to be established whether such an individualized, biomarker-based strategy actually leads to better clinical outcome or whether it is possible to intervene in specific pathophysiological processes.

## 5 Conclusion

This exploratory study investigated the potential of biomarkers measured using the OLINK Cardiovascular III panel to identify associations with clinical events in patients with moderate to severe HF. Our findings suggest that biomarkers most strongly associated with upcoming worsening HF, include established biomarkers such as ST2 and NT-proBNP, as well as other biomarkers such as MMP-2, IGFBP-1 and IGFBP-7. These biomarkers are associated with underlying pathophysiological processes, such as myocardial stress, remodelling and extracellular matrix turnover.

## CRediT authorship contribution statement

**Y. Allach:** Writing – review & editing, Writing – original draft, Visualization, Software, Project administration, Methodology, Investigation, Formal analysis, Data curation. **M. Barry - Loncq de Jong:** Writing – review & editing, Writing – original draft, Visualization, Software, Project administration, Methodology, Investigation, Formal analysis, Data curation. **P.R.D. Clephas:** Writing – review & editing, Visualization, Software, Methodology, Investigation, Formal analysis, Data curation. **S. Abou Kamar:** Writing – review & editing, Visualization, Software, Methodology, Investigation, Formal analysis. **H.P. Brunner-La Rocca:** Writing – review & editing, Resources. **M.L. Handoko:** Writing – review & editing, Resources. **V.P. van Halm:** Writing – review & editing, Resources. **W.E.M. Kok:** Writing – review & editing, Resources. **F.W. Asselbergs:** Writing – review & editing, Resources. **R.R.J. van Kimmenade:** Writing – review & editing, Resources. **S.L.M.A. Beeres:** Writing – review & editing, Resources. **M. Rienstra:** Writing – review & editing, Resources. **M.K. Szymanski:** Writing – review & editing, Resources. **R.A. de Boer:** Writing – review & editing, Resources. **I. Kardys:** Writing – review & editing, Supervision, Methodology, Funding acquisition, Formal analysis, Conceptualization. **J.J. Brugts:** Writing – review & editing, Supervision, Resources, Methodology, Investigation, Funding acquisition, Data curation, Conceptualization.

## Funding

The BioMEMS study is an investigator-initiated study. CRO and Sponsor is the ErasmusMC. The study is embedded in a randomised clinical trial (MONITOR-HF) funded by the Dutch Ministry of Health (conditional coverage program for innovative care). The study is designed, conducted, analysed and reported completely independent of the manufacturer. This work was supported by the Netherlands Heart Institute (NLHI), which provided the NLHI Collaborator grant 2023 that funded the BioMEMS study (YA/JJB). Part of the program is also funded by the Jaap Schouten Foundation (MBL/IK).

## Declaration of competing interest

The authors declare the following financial interests/personal relationships which may be considered as potential competing interests: Jasper J. Brugts and Youssra Allach report financial support was provided by Netherlands Heart Institute. Mylene Barry Loncq de Jong and Isabella Kardys reports financial support was provided by Jaap Schouten Foundation.

H.P. Brunner-la Rocca reports a relationship with Vifor Pharma Inc that includes: consulting or advisory, funding grants, and speaking and lecture fees. H.P. Brunner-la Rocca reports a relationship with Roche Diagnostics that includes: consulting or advisory, funding grants, and speaking and lecture fees. H.P. Brunner-la Rocca reports a relationship with Novartis that includes: consulting or advisory and speaking and lecture fees. H.P. Brunner-la Rocca reports a relationship with Boehringer Ingelheim that includes: consulting or advisory and speaking and lecture fees. M.L. Handoko reports a relationship with Dutch heart foundation that includes: funding grants. M.L. Handoko reports a relationship with Netherlands CardioVascular Research Initiative that includes: funding grants. M.L. Handoko reports a relationship with Vifor Pharma Inc that includes: consulting or advisory, funding grants, and speaking and lecture fees. M.L. Handoko reports a relationship with Boehringer Ingelheim that includes: consulting or advisory, funding grants, and speaking and lecture fees. M.L. Handoko reports a relationship with Daiichi Sankyo Inc that includes: consulting or advisory and speaking and lecture fees. M.L. Handoko reports a relationship with AstraZeneca that includes: consulting or advisory and speaking and lecture fees. M.L. Handoko reports a relationship with Bayer Corporation that includes: consulting or advisory and speaking and lecture fees. M.L. Handoko reports a relationship with MSD that includes: consulting or advisory and speaking and lecture fees. M.L. Handoko reports a relationship with Abbott that includes: consulting or advisory and speaking and lecture fees. M.L. Handoko reports a relationship with Quin Medical that includes: consulting or advisory and speaking and lecture fees. W.E.M. Kok reports a relationship with Novartis that includes: speaking and lecture fees. R. van Kimmenade reports a relationship with Dutch Heart Foundation that includes: funding grants. R. van Kimmenade reports a relationship with Netherlands Heart Institute that includes: funding grants. R. van Kimmenade reports a relationship with Novartis Pharma that includes: consulting or advisory. R. van Kimmenade reports a relationship with Bayer Corporation that includes: consulting or advisory. R. van Kimmenade reports a relationship with Roche Diagnostics that includes: travel reimbursement. R. van Kimmenade has a leadership or fiduciary role in other board society in Nuceus ESC WGPAAD. M. Rienstra is conducted in collaboration with and supported by the Dutch CardioVascular Alliance, 01-002-2022-0118 EmbRACE. S.L.M.A. Beeres reports a relationship with Boston Scientific Corporation that includes: consulting or advisory and speaking and lecture fees. S.L.M.A. Beeres reports a relationship with Medtronic Trading NL BV that includes: consulting or advisory and speaking and lecture fees. S.L.M.A. Beeres reports a relationship with Boehringer Ingelheim that includes: consulting or advisory and speaking and lecture fees. M. Rienstra reports a relationship with Dutch Heart Foundation that includes: funding grants. M. Rienstra reports a relationship with ZonMW that includes: funding grants. M. Rienstra reports a relationship with Netherland Cardiovascular Research Initiative (Dutch Heart Foundation) that includes: funding grants. M. Rienstra reports a relationship with Top Sector Life Sciences & Health that includes: funding grants. M. Rienstra reports a relationship with European Union Horizon 2020 research and innovation programme that includes: funding grants. M. Rienstra reports a relationship with Bayer Corporation that includes: consulting or advisory. M. Rienstra reports a relationship with InCarda Therapeutics, Inc. that includes: consulting or advisory. M. Rienstra reports a relationship with Novartis that includes: consulting or advisory. M. Rienstra reports a relationship with Daiichi Sankyo Inc that includes: speaking and lecture fees. M. Rienstra reports a relationship with Pfizer that includes: speaking and lecture fees. R.A. de Boer reports a relationship with AstraZeneca that includes: funding grants and speaking and lecture fees. R.A. de Boer reports a relationship with Abbott that includes: funding grants, speaking and lecture fees and travel reimbursement. R.A. de Boer reports a relationship with Boehringer Ingelheim that includes: funding grants. R.A. de Boer reports a relationship with Cardior Pharmaceuticals that includes: funding grants and speaking and lecture fees. R.A. de Boer reports a relationship with Novo Nordisk Inc that includes: funding grants, speaking and lecture fees and travel reimbursement. R.A. de Boer reports a relationship with Roche that includes: funding grants and speaking and lecture fees. R.A. de Boer reports a relationship with Bayer Corporation that includes: speaking and lecture fees. R.A. de Boer reports a relationship with Bristol Myers Squibb Co that includes: speaking and lecture fees. R.A. de Boer reports a relationship with Zoll that includes: speaking and lecture fees. I. Kardys reports a relationship with Olink Proteomics that includes: travel reimbursement. I. Kardys reports a relationship with SomaLogic that includes: travel reimbursement. J.J. Brugts reports an independent research grant for ISS (Abbott, Astra Zeneca, Boehringer, Novartis) paid to the Institute and has had invited speaker engagements or advisory boards with Astra Zeneca, Abbott, Bayer, Boehringer, Edwards LifeSciences, Novartis, Vifor and Zoll in the past 5 years. If there are other authors, they declare that they have no known competing financial interests or personal relationships that could have appeared to influence the work reported in this paper.
