# Supplementary material for: Pathophysiological analysis of serially measured plasma biomarkers, remote monitoring and clinical events, in symptomatic patients with moderate to severe chronic heart failure
Source: Int J Cardiol Heart Vasc. 2026 Jul 11;65:101970. doi: 10.1016/j.ijcha.2026.101970 (PMC13377489; doi:10.1016/j.ijcha.2026.101970)
Supplement: Supplementary file 1 — Supplementary material [file mmc1.docx]

**Supplementary material**

**Supplementary table 1 – Baseline characteristics of *all patients (overall study population).*** Data are n (%) or median (IQR). All p values for differences between randomised groups were non-significant. All analyses were based on the intention-to-treat principle. eGFR=estimated glomerular filtration rate. NT-proBNP=N-terminal pro-B natriuretic peptide.

|  | **CardioMEMS (n=176)** | **Standard care (n=172)** |
| --- | --- | --- |
| Age (median (IQR)) | 69 (61–75) | 70 (61–75) |
| Male sex (n, %) | 138 (78·4%) | 125 (72·7%) |
| Previous myocardial infarction (n, (%)) | 81 (46·0%) | 65 (37·8%) |
| Diabetes (n, (%)) | 66 (37·5%) | 68 (39·5%) |
| Cerebrovascular accident or transient ischaemic attack (n, (%)) | 29 (16·5%) | 39 (22·7%) |
| Atrial fibrillation (n, (%)) | 100 (56·8%) | 81 (47·1%) |
| Hypertension (n, (%)) | 102 (58·0%) | 98 (57·0%) |
| eGFR, mL/min (median (IQR)) | 48 (35–60) | 48 (38–63) |
| Body mass index, kg/m2 (median (IQR)) | 27·2 (24·4–31·6) | 26·8 (24·1–31·0) |
| Left ventricular ejection fraction <40% (n, (%)) | 127 (72.7%) | 123 (71·5%) |
| Heart failure etiology = non-ischemic (n, %) | 83 (47.2%) | 91 (52.9%) |
| Years since heart failure diagnosis (median [IQR]) | 3·4 (0·8–8·3) | 3·8 (0·9–8·7) |
| Months since last heart failure hospitalisation (median [IQR]) | 3·6 (1·2–6·4) | 3·4 (1·6–6·7) |
| Beta blockers (n (% yes)) | 150 (85·2%) | 142 (82·6%) |
| Renin-angiotensin-aldosterone system inhibitor (n (% yes)) | 154 (87·5%) | 147 (85·5%) |
| Mineralocorticoid receptor antagonist (n (% yes)) | 143 (81·3%) | 144 (83·7%) |
| SGLT2 inhibitor (n (% yes)) | 12 (6·8%) | 21 (12·2%) |
| Diuretics (n (% yes)) | 11 (6·3%) | 10 (5·8%) |

**Supplementary table 2 - The relationship between serially measured blood biomarkers and the composite study endpoint, assessed using joint modelling.** This model corrected for age, sex, study arm, duration of HF, hypertension, eGFR, and the use of GDMT at baseline and was adjusted for multiple testing (Benjamini Hochberg).

| **Biomarker** | **Hazard ratio** | **95% confidence interval** | | | **P-value** |
| --- | --- | --- | --- | --- | --- |
|  |  | **Lower limit** | | **Upper limit** |  |
| ALCAM | 1,73 | 1,21 | 2,59 | | 0,09 |
| **APN** | **1,54** | **1,20** | **2,00** | | **0,02** |
| AXL | 1,67 | 1,20 | 2,39 | | 0,13 |
| AZU1 | 1,30 | 0,79 | 2,14 | | 0,87 |
| BLM hydrolase | 1,40 | 1,01 | 2,00 | | 0,87 |
| CASP-3 | 0,75 | 0,53 | 1,05 | | 0,87 |
| CCL15 | 1,51 | 1,14 | 2,05 | | 0,17 |
| CCL16 | 1,39 | 1,07 | 1,86 | | 0,60 |
| CCL24 | 1,07 | 0,87 | 1,32 | | 0,87 |
| CD163 | 1,45 | 1,10 | 1,95 | | 0,45 |
| CD93 | 1,85 | 1,25 | 2,79 | | 0,05 |
| CDH5 | 1,44 | 1,05 | 1,96 | | 0,87 |
| CHI3L1 | 1,36 | 1,04 | 1,79 | | 0,87 |
| CHIT1 | 0,94 | 0,81 | 1,12 | | 0,87 |
| CNTN1 | 1,14 | 0,88 | 1,50 | | 0,87 |
| COL1A1 | 1,24 | 0,93 | 1,71 | | 0,87 |
| CPA1 | 1,17 | 0,91 | 1,52 | | 0,87 |
| CPB1 | 1,06 | 0,82 | 1,35 | | 0,87 |
| CSTB | 1,62 | 1,18 | 2,28 | | 0,10 |
| CTSD | 1,57 | 1,16 | 2,18 | | 0,14 |
| CTSZ | 1,06 | 0,85 | 1,40 | | 0,87 |
| CXCL16 | 1,82 | 1,21 | 2,89 | | 0,18 |
| DLK1 | 1,17 | 0,92 | 1,48 | | 0,87 |
| EGFR | 1,32 | 0,93 | 2,10 | | 0,87 |
| Ep-CAM | 1,12 | 0,86 | 1,43 | | 0,87 |
| EPHB4 | 1,50 | 1,07 | 2,12 | | 0,78 |
| FABP4 | 1,46 | 1,09 | 1,96 | | 0,48 |
| FAS | 1,17 | 0,91 | 1,52 | | 0,87 |
| Gal-3 | 1,25 | 0,89 | 1,88 | | 0,87 |
| Gal-4 | 1,11 | 0,84 | 1,48 | | 0,87 |
| **GDF-15** | **1,69** | **1,33** | **2,15** | | **0,01** |
| GP6 | 0,67 | 0,45 | 0,97 | | 0,87 |
| GRN | 1,53 | 1,06 | 2,36 | | 0,83 |
| ICAM-2 | 1,74 | 1,28 | 2,46 | | 0,07 |
| **IGFBP-1** | **2,30** | **1,64** | **3,33** | | **<0,001** |
| **IGFBP-2** | **1,64** | **1,23** | **2,20** | | **0,01** |
| **IGFBP-7** | **2,47** | **1,81** | **3,47** | | **<0,001** |
| IL-17RA | 1,38 | 1,08 | 1,76 | | 0,55 |
| IL-18BP | 1,45 | 1,04 | 2,07 | | 0,87 |
| **IL-1RT1** | **1,89** | **1,39** | **2,61** | | **<0,001** |
| IL-1RT2 | 1,34 | 1,03 | 1,75 | | 0,87 |
| IL2-RA | 1,07 | 0,91 | 1,34 | | 0,87 |
| IL-6RA | 1,45 | 1,10 | 1,93 | | 0,64 |
| ITGB2 | 1,16 | 0,87 | 1,57 | | 0,87 |
| JAM-A | 1,10 | 0,75 | 1,64 | | 0,87 |
| KLK6 | 1,46 | 1,08 | 2,04 | | 0,78 |
| LDL receptor | 0,71 | 0,54 | 0,92 | | 0,47 |
| LTBR | 1,55 | 1,11 | 2,21 | | 0,65 |
| MB | 1,40 | 1,03 | 1,95 | | 0,87 |
| MCP1 | 1,17 | 0,85 | 1,62 | | 0,87 |
| MEPE | 1,08 | 0,81 | 1,45 | | 0,87 |
| **MMP-2** | **2,64** | **1,90** | **3,84** | | **<0,001** |
| **MMP-3** | **2,15** | **1,58** | **3,01** | | **<0,001** |
| MMP-9 | 1,12 | 0,79 | 1,60 | | 0,87 |
| MPO | 1,26 | 0,92 | 1,75 | | 0,87 |
| **Notch 3** | **2,06** | **1,51** | **2,84** | | **<0,001** |
| **NT-proBNP** | **2,07** | **1,61** | **2,70** | | **<0,001** |
| OPG | 1,37 | 1,05 | 1,81 | | 0,87 |
| **OPN** | **2,04** | **1,43** | **2,96** | | **<0,001** |
| PAI | 1,13 | 0,86 | 1,49 | | 0,87 |
| PCSK9 | 0,84 | 0,60 | 1,15 | | 0,87 |
| PDGF subunit A | 0,76 | 0,57 | 1,01 | | 0,87 |
| PECAM-1 | 1,15 | 0,74 | 1,84 | | 0,87 |
| PGLYRP1 | 1,24 | 0,95 | 1,68 | | 0,87 |
| PI3 | 1,11 | 0,88 | 1,41 | | 0,87 |
| PLC | 1,59 | 1,10 | 2,40 | | 0,55 |
| PON3 | 0,79 | 0,62 | 1,00 | | 0,87 |
| PRTN3 | 1,37 | 1,00 | 1,89 | | 0,87 |
| PSP-D | 1,39 | 1,10 | 1,77 | | 0,30 |
| RARRES2 | 1,08 | 0,92 | 1,35 | | 0,87 |
| RETN | 1,40 | 1,05 | 1,93 | | 0,87 |
| **SCGB3A2** | **1,51** | **1,21** | **1,87** | | **0,01** |
| SELE | 1,27 | 1,00 | 1,63 | | 0,87 |
| SELP | 0,98 | 0,73 | 1,33 | | 0,87 |
| SHPS1 | 1,38 | 1,05 | 1,85 | | 0,87 |
| **ST2** | **2,65** | **1,98** | **3,59** | | **<0,001** |
| TFF3 | 1,34 | 1,02 | 1,76 | | 0,87 |
| TFPI | 1,24 | 0,97 | 1,67 | | 0,87 |
| TIMP4 | 1,53 | 1,17 | 2,00 | | 0,13 |
| **TNF-R1** | **2,06** | **1,43** | **3,07** | | **<0,001** |
| TNF-R2 | 1,31 | 1,03 | 1,63 | | 0,87 |
| TNFRSF10C | 1,58 | 1,20 | 2,11 | | 0,06 |
| TNFRSF14 | 1,39 | 0,97 | 2,03 | | 0,87 |
| **TNFSF13B** | **1,60** | **1,23** | **2,09** | | **0,04** |
| t-PA | 1,57 | 1,09 | 2,28 | | 0,75 |
| **TR** | **1,80** | **1,36** | **2,41** | | **0,01** |
| T-RAP | 0,78 | 0,60 | 1,00 | | 0,87 |
| uPA | 1,55 | 1,15 | 2,15 | | 0,24 |
| **U-PAR** | **2,18** | **1,49** | **3,36** | | **0,01** |
| vWF | 1,46 | 0,97 | 2,30 | | 0,87 |

**Supplementary table 3 - The relationship between serially measured blood biomarkers and the composite study endpoint, assessed using joint modelling.** This model corrected for age, sex, study arm, duration of HF, hypertension, eGFR, the use of GDMT at baseline, AF, LVEF and BMI and was adjusted for multiple testing (Benjamini Hochberg).

| **Biomarker** | **Hazard ratio** | **95% confidence interval** | | **P-value** |
| --- | --- | --- | --- | --- |
|  |  | **Lower limit** | **Upper limit** |  |
| ALCAM | 1,73 | 1,20 | 2,58 | 0,09 |
| APN | 1,52 | 1,17 | 1,96 | 0,09 |
| AXL | 1,67 | 1,19 | 2,41 | 0,12 |
| AZU1 | 1,40 | 0,83 | 2,37 | 0,84 |
| BLM hydrolase | 1,42 | 1,02 | 2,07 | 0,84 |
| CASP-3 | 0,74 | 0,51 | 1,05 | 0,84 |
| CCL15 | 1,49 | 1,11 | 2,01 | 0,39 |
| CCL16 | 1,36 | 1,06 | 1,81 | 0,83 |
| CCL24 | 1,08 | 0,87 | 1,33 | 0,84 |
| CD163 | 1,47 | 1,11 | 1,98 | 0,35 |
| **CD93** | **1,86** | **1,22** | **2,92** | **0,04** |
| CDH5 | 1,42 | 1,03 | 1,97 | 0,84 |
| CHI3L1 | 1,36 | 1,05 | 1,79 | 0,84 |
| CHIT1 | 0,92 | 0,79 | 1,10 | 0,84 |
| CNTN1 | 1,14 | 0,87 | 1,50 | 0,84 |
| COL1A1 | 1,25 | 0,93 | 1,73 | 0,84 |
| CPA1 | 1,17 | 0,90 | 1,53 | 0,84 |
| CPB1 | 1,05 | 0,82 | 1,35 | 0,84 |
| CSTB | 1,65 | 1,17 | 2,40 | 0,26 |
| CTSD | 1,63 | 1,18 | 2,28 | 0,08 |
| CTSZ | 1,05 | 0,84 | 1,38 | 0,84 |
| CXCL16 | 1,82 | 1,23 | 2,84 | 0,13 |
| DLK1 | 1,17 | 0,93 | 1,48 | 0,84 |
| EGFR | 1,31 | 0,92 | 2,08 | 0,84 |
| Ep-CAM | 1,10 | 0,85 | 1,42 | 0,84 |
| EPHB4 | 1,49 | 1,04 | 2,17 | 0,84 |
| **FABP4** | 1,76 | 1,26 | 2,44 | 0,06 |
| FAS | 1,17 | 0,90 | 1,53 | 0,84 |
| Gal-3 | 1,24 | 0,87 | 1,87 | 0,84 |
| Gal-4 | 1,06 | 0,80 | 1,40 | 0,84 |
| **GDF-15** | **1,67** | **1,30** | **2,14** | **0,02** |
| GP6 | 0,66 | 0,43 | 0,95 | 0,84 |
| GRN | 1,55 | 1,06 | 2,45 | 0,84 |
| ICAM-2 | 1,74 | 1,25 | 2,45 | 0,05 |
| **IGFBP-1** | **2,70** | **1,81** | **4,27** | **<0,001** |
| **IGFBP-2** | **1,73** | **1,24** | **2,47** | **0,03** |
| **IGFBP-7** | **2,48** | **1,80** | **3,45** | **<0,001** |
| IL-17RA | 1,36 | 1,06 | 1,74 | 0,75 |
| IL-18BP | 1,42 | 1,01 | 2,08 | 0,84 |
| **IL-1RT1** | **1,87** | **1,34** | **2,61** | **<0,001** |
| IL-1RT2 | 1,34 | 1,02 | 1,76 | 0,84 |
| IL2-RA | 1,07 | 0,91 | 1,35 | 0,84 |
| IL-6RA | 1,42 | 1,07 | 1,89 | 0,83 |
| ITGB2 | 1,18 | 0,88 | 1,61 | 0,84 |
| JAM-A | 1,06 | 0,69 | 1,65 | 0,84 |
| KLK6 | 1,41 | 1,03 | 1,99 | 0,84 |
| LDL receptor | 0,72 | 0,55 | 0,93 | 0,76 |
| LTBR | 1,54 | 1,08 | 2,26 | 0,84 |
| MB | 1,37 | 1,00 | 1,90 | 0,84 |
| MCP1 | 1,18 | 0,83 | 1,63 | 0,84 |
| MEPE | 1,07 | 0,80 | 1,44 | 0,84 |
| **MMP-2** | **2,72** | **1,87** | **4,03** | **<0,001** |
| **MMP-3** | **2,17** | **1,57** | **3,04** | **<0,001** |
| MMP-9 | 1,21 | 0,86 | 1,74 | 0,84 |
| MPO | 1,33 | 0,96 | 1,87 | 0,84 |
| **Notch 3** | **2,05** | **1,48** | **2,87** | **<0,001** |
| **NT-proBNP** | **2,28** | **1,72** | **3,03** | **<0,001** |
| OPG | 1,33 | 1,00 | 1,78 | 0,84 |
| **OPN** | **2,05** | **1,43** | **2,97** | **<0,001** |
| PAI | 1,21 | 0,90 | 1,63 | 0,84 |
| PCSK9 | 0,84 | 0,60 | 1,15 | 0,84 |
| PDGF subunit A | 0,78 | 0,57 | 1,05 | 0,84 |
| PECAM-1 | 1,13 | 0,70 | 1,86 | 0,84 |
| PGLYRP1 | 1,27 | 0,96 | 1,73 | 0,84 |
| PI3 | 1,08 | 0,85 | 1,39 | 0,84 |
| PLC | 1,65 | 1,11 | 2,51 | 0,39 |
| PON3 | 0,74 | 0,58 | 0,94 | 0,68 |
| PRTN3 | 1,44 | 1,03 | 2,03 | 0,84 |
| PSP-D | 1,37 | 1,07 | 1,76 | 0,68 |
| RARRES2 | 1,08 | 0,91 | 1,39 | 0,84 |
| RETN | 1,44 | 1,05 | 2,04 | 0,84 |
| SCGB3A2 | 1,50 | 1,19 | 1,89 | 0,07 |
| SELE | 1,31 | 1,02 | 1,71 | 0,84 |
| SELP | 0,97 | 0,72 | 1,35 | 0,84 |
| SHPS1 | 1,39 | 1,05 | 1,83 | 0,84 |
| **ST2** | **2,71** | **2,00** | **3,81** | **<0,001** |
| TFF3 | 1,29 | 0,97 | 1,75 | 0,84 |
| TFPI | 1,23 | 0,95 | 1,66 | 0,84 |
| TIMP4 | 1,51 | 1,16 | 1,99 | 0,20 |
| **TNF-R1** | **2,06** | **1,40** | **3,19** | **<0,001** |
| TNF-R2 | 1,31 | 1,02 | 1,64 | 0,84 |
| TNFRSF10C | 1,56 | 1,19 | 2,09 | 0,10 |
| TNFRSF14 | 1,35 | 0,90 | 2,01 | 0,84 |
| **TNFSF13B** | **1,60** | **1,22** | **2,11** | **0,04** |
| t-PA | 1,69 | 1,18 | 2,47 | 0,26 |
| **TR** | **1,85** | **1,39** | **2,49** | **<0,001** |
| T-RAP | 0,76 | 0,59 | 0,99 | 0,84 |
| uPA | 1,61 | 1,16 | 2,31 | 0,23 |
| **U-PAR** | **2,19** | **1,47** | **3,38** | **<0,001** |
| vWF | 1,54 | 0,98 | 2,52 | 0,84 |

**Supplementary table 4 – Subgroup analysis**

**Serially measured blood biomarkers and the composite study *endpoint in patients with either remote hemodynamic monitoring on top of standard of care or patients with standard of care****.* This model corrected for age, sex, duration of HF, hypertension, eGFR, and the use of GDMT at baseline, and was adjusted for multiple testing (Benjamini Hochberg). The last column indicates a p-value for the interaction term for the treatment groups. This was not significant for all biomarkers. NB: splines were not used for the analysis of the following biomarkers: CD93, CDH5, GRN, IL-17RA, IL-1RT1, and NOTCH-3.

| **Remote hemodynamic monitoring** | | | |  |  | **Standard of Care** | |  |  |  |  | **Interaction** |
| --- | --- | --- | --- | --- | --- | --- | --- | --- | --- | --- | --- | --- |
| **Biomarker** | **Hazard ratio** | **95% confidence interval** | | **P-value** |  | **Biomarker** | **Hazard ratio** | **95% confidence interval** | | **P-value** |  | **P-value** |
|  |  | **Lower limit** | **Upper limit** |  |  |  |  | **Lower limit** | **Upper limit** |  |  |  |
| ALCAM | 3,05 | 1,52 | 6,56 | 0,13 |  | ALCAM | 1,40 | 1,00 | 2,20 | 0,96 |  | 0,99 |
| APN | 1,61 | 1,09 | 2,32 | 0,99 |  | APN | 1,36 | 1,01 | 1,88 | 0,96 |  | 0,99 |
| AXL | 1,85 | 1,12 | 3,18 | 0,99 |  | AXL | 1,63 | 1,06 | 2,78 | 0,96 |  | 0,99 |
| AZU1 | 0,77 | 0,31 | 1,83 | 0,99 |  | AZU1 | 1,70 | 0,82 | 4,33 | 0,96 |  | 0,99 |
| BLM hydrolase | 0,89 | 0,54 | 1,50 | 0,99 |  | BLM hydrolase | 1,54 | 1,01 | 2,63 | 0,96 |  | 0,99 |
| CASP-3 | 0,74 | 0,45 | 1,18 | 0,99 |  | CASP-3 | 0,72 | 0,40 | 1,23 | 0,96 |  | 0,99 |
| CCL15 | 1,48 | 0,96 | 2,24 | 0,99 |  | CCL15 | 1,50 | 1,05 | 2,25 | 0,96 |  | 0,99 |
| CCL16 | 1,48 | 0,99 | 2,26 | 0,99 |  | CCL16 | 1,48 | 1,04 | 2,23 | 0,96 |  | 0,99 |
| CCL24 | 1,11 | 0,81 | 1,49 | 0,99 |  | CCL24 | 1,03 | 0,78 | 1,37 | 0,96 |  | 0,99 |
| CD163 | 1,67 | 1,08 | 2,58 | 0,99 |  | CD163 | 1,20 | 0,91 | 1,69 | 0,96 |  | 0,99 |
| CD93 | 1,33 | 0,65 | 2,74 | 0,99 |  | CD93 | 1,33 | 0,65 | 2,74 | 0,96 |  | 0,99 |
| CDH5 | 1,71 | 1,00 | 3,02 | 0,99 |  | CDH5 | 1,71 | 1,00 | 3,02 | 0,96 |  | 0,99 |
| CHI3L1 | 1,51 | 0,98 | 2,35 | 0,99 |  | CHI3L1 | 1,31 | 0,94 | 1,83 | 0,96 |  | 0,99 |
| CHIT1 | 0,85 | 0,70 | 1,06 | 0,99 |  | CHIT1 | 1,07 | 0,83 | 1,42 | 0,96 |  | 0,99 |
| CNTN1 | 1,41 | 0,94 | 2,13 | 0,99 |  | CNTN1 | 1,04 | 0,75 | 1,48 | 0,96 |  | 0,99 |
| COL1A1 | 1,24 | 0,79 | 2,05 | 0,99 |  | COL1A1 | 1,27 | 0,88 | 1,99 | 0,96 |  | 0,99 |
| CPA1 | 1,32 | 0,93 | 1,88 | 0,99 |  | CPA1 | 0,99 | 0,70 | 1,41 | 0,96 |  | 0,99 |
| CPB1 | 1,34 | 0,91 | 1,98 | 0,99 |  | CPB1 | 0,91 | 0,66 | 1,23 | 0,96 |  | 0,99 |
| CSTB | 2,14 | 1,20 | 3,89 | 0,48 |  | CSTB | 1,58 | 1,00 | 2,66 | 0,96 |  | 0,99 |
| CTSD | 1,67 | 1,06 | 2,69 | 0,99 |  | CTSD | 1,59 | 1,05 | 2,60 | 0,96 |  | 0,99 |
| CTSZ | 0,89 | 0,57 | 1,43 | 0,99 |  | CTSZ | 1,12 | 0,87 | 1,60 | 0,96 |  | 0,99 |
| CXCL16 | 1,75 | 1,05 | 3,00 | 0,99 |  | CXCL16 | 2,12 | 1,16 | 4,84 | 0,63 |  | 0,99 |
| DLK1 | 1,14 | 0,83 | 1,56 | 0,99 |  | DLK1 | 1,25 | 0,88 | 1,82 | 0,96 |  | 0,99 |
| EGFR | 1,25 | 0,57 | 2,73 | 0,99 |  | EGFR | 1,30 | 0,92 | 2,13 | 0,96 |  | 0,99 |
| Ep-CAM | 0,97 | 0,66 | 1,40 | 0,99 |  | Ep-CAM | 1,28 | 0,91 | 1,81 | 0,96 |  | 0,99 |
| EPHB4 | 1,37 | 0,84 | 2,19 | 0,99 |  | EPHB4 | 1,74 | 1,01 | 3,41 | 0,96 |  | 0,99 |
| FABP4 | 1,92 | 1,23 | 3,03 | 0,25 |  | FABP4 | 1,36 | 0,91 | 2,05 | 0,96 |  | 0,99 |
| FAS | 1,06 | 0,71 | 1,47 | 0,99 |  | FAS | 1,23 | 0,89 | 1,91 | 0,96 |  | 0,99 |
| Gal-3 | 1,29 | 0,72 | 2,32 | 0,99 |  | Gal-3 | 1,15 | 0,81 | 1,82 | 0,96 |  | 0,99 |
| Gal-4 | 1,15 | 0,75 | 1,75 | 0,99 |  | Gal-4 | 1,05 | 0,72 | 1,54 | 0,96 |  | 0,99 |
| **GDF-15** | **2,14** | **1,45** | **3,19** | **<0,001** |  | GDF-15 | 1,56 | 1,13 | 2,18 | 0,43 |  | 0,99 |
| GP6 | 0,86 | 0,45 | 1,52 | 0,99 |  | GP6 | 0,57 | 0,30 | 0,99 | 0,96 |  | 0,99 |
| GRN | 1,90 | 0,99 | 3,87 | 0,99 |  | GRN | 1,90 | 0,99 | 3,87 | 0,96 |  | 0,99 |
| **ICAM-2** | **3,33** | **1,72** | **7,40** | **<0,001** |  | ICAM-2 | 1,41 | 0,94 | 2,18 | 0,96 |  | 0,99 |
| IGFBP-1 | **2,54** | **1,51** | **4,67** | **0,04** |  | **IGFBP-1** | **2,41** | **1,47** | **4,42** | **0,01** |  | 0,99 |
| IGFBP-2 | 2,02 | 1,30 | 3,17 | 0,12 |  | IGFBP-2 | 1,37 | 0,98 | 2,01 | 0,96 |  | 0,99 |
| **IGFBP-7** | **2,88** | **1,88** | **4,47** | **<0,001** |  | **IGFBP-7** | **2,30** | **1,49** | **3,80** | **0,01** |  | 0,99 |
| IL-17RA | 1,36 | 0,94 | 2,00 | 0,99 |  | IL-17RA | 1,36 | 0,94 | 2,00 | 0,96 |  | 0,99 |
| IL-18BP | 1,46 | 0,86 | 2,49 | 0,99 |  | IL-18BP | 1,40 | 0,95 | 2,28 | 0,96 |  | 0,99 |
| IL-1RT1 | 2,19 | 1,27 | 3,83 | 0,19 |  | IL-1RT1 | 2,19 | 1,27 | 3,83 | 0,19 |  | 0,99 |
| IL-1RT2 | 1,39 | 0,92 | 2,10 | 0,99 |  | IL-1RT2 | 1,30 | 0,93 | 1,93 | 0,96 |  | 0,99 |
| IL2-RA | 1,38 | 0,91 | 2,08 | 0,99 |  | IL-6RA | 1,10 | 0,91 | 1,46 | 0,96 |  | 0,99 |
| IL-6RA | 1,09 | 0,61 | 1,97 | 0,99 |  | IL2-RA | 1,43 | 0,97 | 2,15 | 0,96 |  | 0,99 |
| ITGB2 | 1,40 | 0,87 | 2,32 | 0,99 |  | ITGB2 | 1,03 | 0,72 | 1,55 | 0,96 |  | 0,99 |
| JAM-A | 1,26 | 0,63 | 2,41 | 0,99 |  | JAM-A | 1,02 | 0,58 | 1,82 | 0,96 |  | 0,99 |
| KLK6 | 1,84 | 1,15 | 3,00 | 0,76 |  | KLK6 | 1,35 | 0,96 | 2,08 | 0,96 |  | 0,99 |
| LDL receptor | 0,88 | 0,57 | 1,31 | 0,99 |  | LDL receptor | 0,61 | 0,39 | 0,90 | 0,74 |  | 0,99 |
| LTBR | 1,41 | 0,87 | 2,25 | 0,99 |  | LTBR | 1,84 | 1,13 | 3,26 | 0,96 |  | 0,99 |
| MB | 1,00 | 0,64 | 1,57 | 0,99 |  | MB | 1,82 | 1,13 | 3,23 | 0,81 |  | 0,99 |
| MCP1 | 1,27 | 0,86 | 1,81 | 0,99 |  | MCP1 | 0,97 | 0,54 | 1,70 | 0,96 |  | 0,99 |
| MEPE | 1,10 | 0,72 | 1,69 | 0,99 |  | MEPE | 1,06 | 0,71 | 1,62 | 0,96 |  | 0,99 |
| MMP-2 | 2,20 | 1,34 | 3,77 | 0,21 |  | **MMP-2** | **4,12** | **2,34** | **8,08** | **<0,001** |  | 0,99 |
| MMP-3 | 1,74 | 1,11 | 2,81 | 0,99 |  | **MMP-3** | **2,81** | **1,73** | **4,99** | **<0,001** |  | 0,99 |
| MMP-9 | 0,90 | 0,56 | 1,47 | 0,99 |  | MMP-9 | 1,29 | 0,82 | 2,07 | 0,96 |  | 0,99 |
| MPO | 1,25 | 0,75 | 2,21 | 0,99 |  | MPO | 1,28 | 0,84 | 2,03 | 0,96 |  | 0,99 |
| Notch 3 | 1,95 | 1,20 | 3,17 | 0,66 |  | Notch 3 | 1,95 | 1,20 | 3,17 | 0,68 |  | 0,99 |
| NT-proBNP | 1,83 | 1,23 | 2,75 | 0,16 |  | **NT-proBNP** | **3,01** | **2,06** | **4,52** | **<0,001** |  | 0,99 |
| OPG | 1,54 | 1,00 | 2,42 | 0,99 |  | OPG | 1,39 | 0,97 | 2,07 | 0,96 |  | 0,99 |
| **OPN** | **2,38** | **1,47** | **4,04** | **0,03** |  | OPN | 1,78 | 1,12 | 3,21 | 0,94 |  | 0,99 |
| PAI | 1,04 | 0,68 | 1,54 | 0,99 |  | PAI | 1,15 | 0,79 | 1,71 | 0,96 |  | 0,99 |
| PCSK9 | 1,01 | 0,68 | 1,45 | 0,99 |  | PCSK9 | 0,56 | 0,29 | 1,03 | 0,96 |  | 0,99 |
| PDGF subunit A | 0,92 | 0,61 | 1,34 | 0,99 |  | PDGF subunit A | 0,59 | 0,37 | 0,91 | 0,96 |  | 0,99 |
| PECAM-1 | 1,43 | 0,72 | 2,94 | 0,99 |  | PECAM-1 | 0,98 | 0,51 | 1,87 | 0,96 |  | 0,99 |
| PGLYRP1 | 1,15 | 0,67 | 1,89 | 0,99 |  | PGLYRP1 | 1,29 | 0,94 | 1,90 | 0,96 |  | 0,99 |
| PI3 | 0,98 | 0,70 | 1,38 | 0,99 |  | PI3 | 1,29 | 0,91 | 1,86 | 0,96 |  | 0,99 |
| PLC | 2,10 | 1,06 | 4,39 | 0,99 |  | PLC | 1,62 | 1,06 | 2,69 | 0,96 |  | 0,99 |
| PON3 | 0,92 | 0,57 | 1,47 | 0,99 |  | PON3 | 0,81 | 0,64 | 1,04 | 0,96 |  | 0,99 |
| PRTN3 | 1,26 | 0,77 | 2,01 | 0,99 |  | PRTN3 | 1,39 | 0,90 | 2,26 | 0,96 |  | 0,99 |
| PSP-D | 1,41 | 0,97 | 2,06 | 0,99 |  | PSP-D | 1,50 | 1,08 | 2,11 | 0,94 |  | 0,99 |
| RARRES2 | 0,69 | 0,28 | 1,69 | 0,99 |  | RARRES2 | 1,11 | 0,93 | 1,48 | 0,96 |  | 0,99 |
| RETN | 1,76 | 1,11 | 2,82 | 0,99 |  | RETN | 1,24 | 0,91 | 1,84 | 0,96 |  | 0,99 |
| SCGB3A2 | 1,62 | 1,16 | 2,21 | 0,53 |  | SCGB3A2 | 1,52 | 1,10 | 2,10 | 0,68 |  | 0,99 |
| SELE | 2,19 | 1,27 | 3,85 | 0,32 |  | SELE | 1,11 | 0,89 | 1,44 | 0,96 |  | 0,99 |
| SELP | 0,98 | 0,59 | 1,62 | 0,99 |  | SELP | 0,96 | 0,68 | 1,44 | 0,96 |  | 0,99 |
| SHPS1 | 2,01 | 1,17 | 3,57 | 0,62 |  | SHPS1 | 1,30 | 0,90 | 1,94 | 0,96 |  | 0,99 |
| **ST2** | **2,43** | **1,66** | **3,72** | **<0,001** |  | **ST2** | **3,34** | **2,02** | **6,46** | **<0,001** |  | 0,99 |
| TFF3 | 1,33 | 0,88 | 1,99 | 0,99 |  | t-PA | 1,64 | 1,01 | 2,77 | 0,96 |  | 0,99 |
| TFPI | 1,81 | 1,14 | 2,94 | 0,99 |  | TFF3 | 1,32 | 0,94 | 1,88 | 0,96 |  | 0,99 |
| TIMP4 | 1,59 | 1,07 | 2,34 | 0,99 |  | TFPI | 1,12 | 0,89 | 1,51 | 0,96 |  | 0,99 |
| TNF-R1 | 1,91 | 1,21 | 3,14 | 0,54 |  | TIMP4 | 1,60 | 1,11 | 2,34 | 0,86 |  | 0,99 |
| TNF-R2 | 1,38 | 0,95 | 1,90 | 0,99 |  | TNF-R1 | 2,20 | 1,29 | 4,09 | 0,28 |  | 0,99 |
| TNFRSF10C | 1,32 | 0,92 | 1,94 | 0,99 |  | TNF-R2 | 1,66 | 0,96 | 3,08 | 0,96 |  | 0,99 |
| TNFRSF14 | 1,33 | 0,80 | 2,21 | 0,99 |  | **TNFRSF10C** | **2,08** | **1,33** | **3,51** | **0,01** |  | 0,99 |
| TNFSF13B | 1,28 | 0,88 | 1,81 | 0,99 |  | TNFRSF14 | 1,50 | 0,87 | 2,68 | 0,96 |  | 0,99 |
| t-PA | 1,49 | 0,81 | 2,79 | 0,99 |  | TNFSF13B | 1,73 | 1,15 | 2,79 | 0,48 |  | 0,99 |
| TR | 1,38 | 0,86 | 2,20 | 0,99 |  | **TR** | **2,02** | **1,41** | **3,06** | **<0,001** |  | 0,99 |
| T-RAP | 0,70 | 0,47 | 1,03 | 0,99 |  | T-RAP | 0,76 | 0,52 | 1,08 | 0,96 |  | 0,99 |
| uPA | 1,66 | 1,12 | 2,43 | 0,78 |  | U-PAR | 2,46 | 1,34 | 5,30 | 0,11 |  | 0,99 |
| U-PAR | 1,85 | 1,08 | 3,09 | 0,99 |  | uPA | 1,40 | 0,95 | 2,35 | 0,96 |  | 0,99 |
| vWF | 1,44 | 0,74 | 2,86 | 0,99 |  | vWF | 1,49 | 0,86 | 3,16 | 0,96 |  | 0,99 |

**Supplementary table 5 – Sensitivity analysis using the expanded linear mixed-effects model**

**Serially measured blood biomarkers and the composite study endpoint, assessed using joint modelling**. This model corrected for age, sex, study arm, duration of HF, hypertension, eGFR, and the use of GDMT at baseline in the Cox-model and included an expanded linear mixed-effects model adjusting for age, sex, study arm , duration of HF, hypertension, eGFR, use of GDMT, AF, LVEF and BMI. splines were not used for the analysis of the biomarker IL-1RT1. The whole model was adjusted for multiple testing (Benjamini Hochberg).

| **Biomarker** | **Hazard ratio** | **95% confidence interval** | | **P-value** |
| --- | --- | --- | --- | --- |
|  |  | **Lower limit** | **Upper limit** |  |
| ALCAM | 1,79 | 1,22 | 2,72 | 0,10 |
| **APN** | **1,54** | **1,18** | **2,00** | **0,02** |
| AXL | 1,74 | 1,24 | 2,66 | 0,06 |
| AZU1 | 1,38 | 0,78 | 2,45 | 0,87 |
| BLM hydrolase | 1,42 | 1,02 | 2,08 | 0,87 |
| CASP-3 | 0,74 | 0,51 | 1,06 | 0,87 |
| CCL15 | 1,53 | 1,13 | 2,08 | 0,27 |
| CCL16 | 1,35 | 1,04 | 1,81 | 0,87 |
| CCL24 | 1,07 | 0,87 | 1,32 | 0,87 |
| CD163 | 1,51 | 1,13 | 2,06 | 0,25 |
| CD93 | 1,89 | 1,24 | 2,99 | 0,06 |
| CDH5 | 1,45 | 1,04 | 2,03 | 0,87 |
| CHI3L1 | 1,37 | 1,04 | 1,82 | 0,87 |
| CHIT1 | 0,92 | 0,79 | 1,11 | 0,87 |
| CNTN1 | 1,15 | 0,86 | 1,53 | 0,87 |
| COL1A1 | 1,27 | 0,95 | 1,77 | 0,87 |
| CPA1 | 1,17 | 0,89 | 1,54 | 0,87 |
| CPB1 | 1,05 | 0,80 | 1,36 | 0,87 |
| CSTB | 1,70 | 1,20 | 2,49 | 0,08 |
| CTSD | 1,69 | 1,22 | 2,44 | 0,06 |
| CTSZ | 1,09 | 0,85 | 1,47 | 0,87 |
| CXCL16 | 1,87 | 1,24 | 2,95 | 0,16 |
| DLK1 | 1,17 | 0,93 | 1,49 | 0,87 |
| EGFR | 1,29 | 0,90 | 2,09 | 0,87 |
| Ep-CAM | 1,10 | 0,85 | 1,43 | 0,87 |
| EPHB4 | 1,57 | 1,08 | 2,35 | 0,76 |
| **FABP4** | **1,81** | **1,29** | **2,56** | **0,04** |
| FAS | 1,19 | 0,91 | 1,57 | 0,87 |
| Gal-3 | 1,24 | 0,88 | 1,85 | 0,87 |
| Gal-4 | 1,08 | 0,81 | 1,46 | 0,87 |
| **GDF-15** | **1,71** | **1,32** | **2,22** | **<0,001** |
| GP6 | 0,67 | 0,45 | 0,98 | 0,87 |
| GRN | 1,57 | 1,06 | 2,52 | 0,87 |
| **ICAM-2** | **1,78** | **1,27** | **2,52** | **0,02** |
| **IGFBP-1** | **3,19** | **1,94** | **5,80** | **<0,001** |
| **IGFBP-2** | **1,80** | **1,29** | **2,59** | **0,01** |
| **IGFBP-7** | **2,53** | **1,83** | **3,62** | **<0,001** |
| IL-17RA | 1,38 | 1,07 | 1,79 | 0,74 |
| IL-18BP | 1,48 | 1,03 | 2,20 | 0,87 |
| **IL-1RT1** | **2,15** | **1,48** | **3,27** | **<0,001** |
| IL-1RT2 | 1,35 | 1,03 | 1,78 | 0,87 |
| IL-6RA | 1,08 | 0,90 | 1,38 | 0,87 |
| IL2-RA | 1,44 | 1,07 | 1,94 | 0,84 |
| ITGB2 | 1,20 | 0,88 | 1,68 | 0,87 |
| JAM-A | 1,11 | 0,72 | 1,69 | 0,87 |
| KLK6 | 1,42 | 1,02 | 2,00 | 0,87 |
| LDL receptor | 0,70 | 0,53 | 0,93 | 0,84 |
| LTBR | 1,60 | 1,10 | 2,35 | 0,64 |
| MB | 1,42 | 1,01 | 2,02 | 0,87 |
| MCP1 | 1,19 | 0,84 | 1,69 | 0,87 |
| MEPE | 1,07 | 0,79 | 1,44 | 0,87 |
| **MMP-2** | **2,81** | **1,92** | **4,35** | **<0,001** |
| **MMP-3** | **2,20** | **1,60** | **3,10** | **<0,001** |
| MMP-9 | 1,24 | 0,86 | 1,83 | 0,87 |
| MPO | 1,34 | 0,94 | 1,94 | 0,87 |
| **Notch 3** | **2,09** | **1,51** | **2,93** | **<0,001** |
| **NT-proBNP** | **2,32** | **1,73** | **3,15** | **<0,001** |
| OPG | 1,39 | 1,02 | 1,92 | 0,87 |
| **OPN** | **2,14** | **1,47** | **3,20** | **0,01** |
| PAI | 1,23 | 0,91 | 1,67 | 0,87 |
| PCSK9 | 0,84 | 0,59 | 1,15 | 0,87 |
| PDGF subunit A | 0,78 | 0,58 | 1,05 | 0,87 |
| PECAM-1 | 1,17 | 0,70 | 1,93 | 0,87 |
| PGLYRP1 | 1,31 | 0,98 | 1,84 | 0,87 |
| PI3 | 1,08 | 0,85 | 1,39 | 0,87 |
| PLC | 1,70 | 1,15 | 2,54 | 0,18 |
| PON3 | 0,73 | 0,58 | 0,94 | 0,74 |
| PRTN3 | 1,44 | 1,02 | 2,04 | 0,87 |
| PSP-D | 1,37 | 1,08 | 1,76 | 0,64 |
| RARRES2 | 1,09 | 0,92 | 1,43 | 0,87 |
| RETN | 1,46 | 1,06 | 2,06 | 0,87 |
| **SCGB3A2** | **1,53** | **1,21** | **1,93** | **0,01** |
| SELE | 1,33 | 1,04 | 1,74 | 0,87 |
| SELP | 0,97 | 0,71 | 1,39 | 0,87 |
| SHPS1 | 1,39 | 1,06 | 1,87 | 0,84 |
| **ST2** | **2,82** | **2,03** | **4,05** | **<0,001** |
| t-PA | 1,72 | 1,18 | 2,54 | 0,22 |
| TFF3 | 1,33 | 0,99 | 1,81 | 0,87 |
| TFPI | 1,24 | 0,95 | 1,69 | 0,87 |
| TIMP4 | 1,51 | 1,16 | 1,98 | 0,11 |
| **TNF-R1** | **2,14** | **1,46** | **3,21** | **<0,001** |
| TNF-R2 | 1,32 | 1,02 | 1,65 | 0,87 |
| **TNFRSF10C** | **1,58** | **1,20** | **2,15** | **0,02** |
| TNFRSF14 | 1,41 | 0,93 | 2,15 | 0,87 |
| **TNFSF13B** | **1,63** | **1,24** | **2,15** | **0,05** |
| **TR** | **1,89** | **1,41** | **2,60** | **<0,001** |
| T-RAP | 0,76 | 0,59 | 1,00 | 0,87 |
| **U-PAR** | **2,27** | **1,49** | **3,57** | **0,01** |
| uPA | 1,63 | 1,19 | 2,30 | 0,06 |
| vWF | 1,60 | 1,00 | 2,76 | 0,87 |

**Supplementary figure 1 – General overview figure of *on the explorative analyses of* biomarkers associated with mPAP and biomarkers associated with clinical events^[[1]](#footnote-1)^.** 13 biomarkers were consistently significantly associated with mPAP and 15 were significantly associated with clinical events. *For an informative overview, the* overlap of both patterns is shown in the figure.


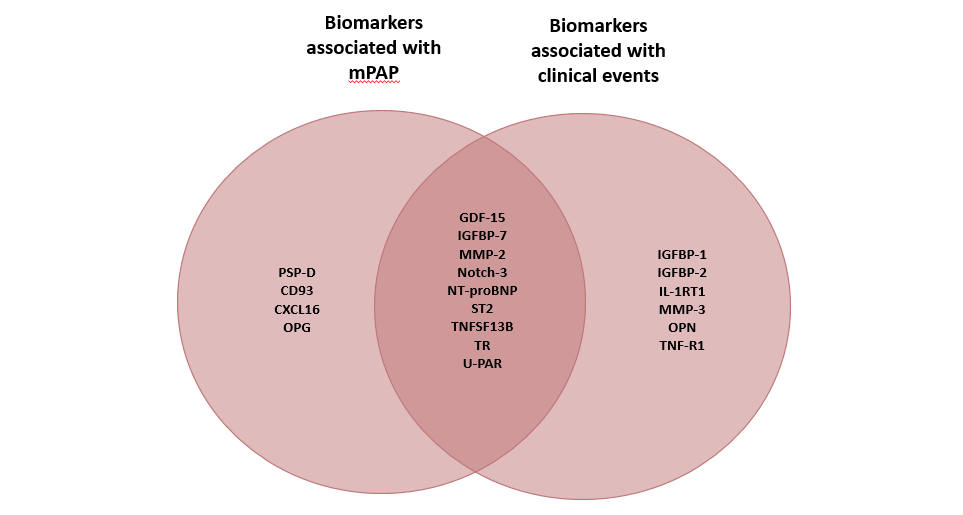


1. Barry-Loncq De Jong M, Allach Y, Abou Kamar S, Clephas PRD, Brunner-La Rocca H-P, Handoko ML, et al. The Association Between Serially Measured Circulating Biomarker Patterns and Pulmonary Artery Pressures Measured by Invasive Hemodynamic Monitoring. J Card Fail 2025. [↑](#footnote-ref-1)
